# Laryngeal and Paryngeal Diseases, Mistaken for Phthisis

**Published:** 1854-01

**Authors:** 

**Affiliations:** Edinburgh


### *Laryngeal and Pharyngeal Diseases, mistaken for Phthisis.*

BY PROFESSOR BENNETT, EDINBURGH.

[In the first case related by Dr, Bennett, although no disease or abnormal sound could be detected in the chest, yet the patient, a gentleman, had hawked up form time to time a small clot of blood about the size of a pea. He remarks :]

The origin of the blood in this case appeared to me at that time to be very mysterious. It was not florid. There was no reason to suppose it to be of pulmonary origin. There was nothing in his voice to indicate laryngeal disease. I did not examine the pharynx, not being then aware of the importance which ought to be attached to it. I was consequently left in great doubt as to the origin of the blood, and of the best means of removing anxiety from my patient. My uncertainty, however, was partially dispelled by the following case :

I was requested by an assurance office, in July 1850 to examine the chest of Mr. M., a merchant, aged about 30, who said he labored under no kind of complaint, with the exception of occasional sorethroat, and expectoration of mucus tinged with blood. He was tolerably stout, took long walks without uneasiness, and suffered from no difficulty of respiration or from cough. Repeated examination of his chest failed to elicit any physical sign indicative of pulmonary disease. I therefore certified that his lungs were healthy. In October, 1851, this gentleman called upon me again for advice, under the following circumstances. The soreness of the throat had latterly increased, and considerable cough was induced, after which he spit up mouthfuls of purulent matter, frequently tinged of a red color. He brought me some of this sputum to examine, which consisted of mixed blood and pus, of a dirty brick-red color. Examination of his chest again convinced me that the lungs were unaffected; but in the interval I paid attention to the writings and practice of Doctor Horace Green, of New York ; and I now examined his throat, when the cause of his symptoms were at once apparent. The fauces and upper part of the pharynx were studded over with nodular swellings, varying in size from a pin head to that of a pea. Many of them were bright red and fungoid in character, probably the origin of the extravasated blood, whilst considerable patches of purulent matter adhered to several parts of the mucus membrane. I applied a sponge, saturated with strong solution of nitrae of silver to the affected parts. In three days he returned, having been much relived, when the application was repeated. I have not since seen him.

These two cases convinced me that certain symptoms which have hitherto been considered as indicative of phthisis might have their origin entirely in the fauces, pharynx, and upper part of the larynx. The cough so occasioned with the purulent expectoration, often tinged with blood, frequently so resembles that occasioned by phthisis, as not only to induce alarm in the minds of patients, but frequently to mislead the medical practitioner. I have now met with many such cases, which have been mistaken for phthisis, and which have been treated for that dissase without any effect, until local remedies were applied, when they for the most part disappeared, or became much better.

[In a second case, that of a female aged 25, there were all the symptoms of phthisis present—frequent cough with hemoptysis, copious purulent expectoration, night sweats, and loss of appetite with vomiting. On examining the fauces they were found covered with purulent mucus. The cough was also ascertained to be convulsive, and a ringing sound was heard over the larynx on inspiration. Laryngitis was the disease diagnosed. The solution of nitrate of silver (ʒss. ʒj. of water) was applied to the fauces, and afterwards the sponge was introduced into the larynx, with some degree of benefit. Blisters to the larynx were also applied ; and as the disease seemed to have a syphilitic origin, iodide of potassium and bitter infusions were the internal remedies resorted to This patient, though not cured, was considerably relieved. Dr. Bennett concludes by saying :

The cases now given, with others that might have been adduced, have satisfied me that lessions of the pharynx and larynx ought to occupy the serious attention of the practitioner in all cases of pulmonary diseases, and that the following practical conclusions may be drawn from them :

1st. That not unfrequently diseases, entirely seated in the larynx or pharynx, are mistaken for phthisis pulmonalis.

2d. That even when pulmonary phthisis exists, many of the urgent symptoms are not so much owing to disease in the lungs as to the pharynegeal and laryngal complications.

3d. That a local treatment may not only remove or alleviate these complications, but that, in conjunction with general remedies, it tends in a marked manner to induce arrestment of the pulmonary disease. *Monthly Jounal of Med. Science*

